# *Aeromonas* wound infection in a healthy boy, and wound healing with polarized light

**DOI:** 10.1099/jmmcr.0.005118

**Published:** 2017-10-16

**Authors:** Bart Rutteman, Kristien Borremans, Jan Beckers, Els Devleeschouwer, Sybien Lampmann, Ivo Corthouts, Piet Verlinde

**Affiliations:** ^1^​ Department of Pediatrics, AZ Sint Blasius Dendermonde, Dendermonde, Belgium; ^2^​ Department of Clinical Microbiology, AZ Sint Blasius Dendermonde, Dendermonde, Belgium

**Keywords:** *aeromonas*, wound infection, polarized light

## Abstract

**Introduction.** At emergency departments, history taking is often very brief. We present a case of an *Aeromonas* wound infection, that illustrates the importance of careful history taking. We also report the first successful use of polarized light as additional therapy for healing of this infectious wound.

**Case presentation.** A healthy boy was diagnosed with a wound infection, after a fall onto rocks. At first, it remained unmentioned that there had been contact with ditchwater, so he was treated with amoxicillin-clavulanic acid. Only after the finding of an *Aeromonas* strain in the wound culture, and treatment with a fluoroquinolone, did he recover. Wound healing was aided with the use of polarized light, and with good effect. To our knowledge, this is the first report on the effect of polarized light on the healing of infectious wounds.

**Conclusion.** Careful history taking is essential for adequate empiric therapy when faced with wounds and wound infections. *Aeromonas* infections are associated with water exposure, and should be treated with fluoroquinolones. Polarized light seems to have a good result on healing of infectious wounds.

## Abbreviations

A. veronii, Aeromonas veronii; A. sobria, Aeromonas sobria; A. hydrophila, Aeromonas hydrophila.

## Introduction

Wound infections are frequently seen after penetrating injuries. Empiric treatment usually consists of amoxicillin-clavulanic acid, aimed at the skin colonizers *Streptococcus pyogenes* and *Staphylococcus aureus*.

Here we present a case, where standard empiric treatment proved to be inadequate, because of infection with an amoxicillin-clavulanic acid resistant *Aeromonas* strain.

## Case report

A 12 year old boy let go too soon from a rope swing over water, and fell onto rocks with his knees. He was taken to hospital with a deep wound under his left knee.

In the emergency room he was diagnosed with a penetrating wound of the left lower leg, exposing a part of the proximal tibia. The wound was sutured subcutaneously and cutaneously, and he was given amoxicillin-clavulanic acid (dosed 875/125 mg twice daily, for a weight of 45 kg).

The day afterwards (day 1), he returned to the emergency ward with photophobia, headache, nausea and fever (38.5 °C). Repeated history made clear that he had fallen about 2–3 m, but not directly on his head. There had been no loss of consciousness nor post-traumatic vomiting. Physical examination showed no signs of sepsis, a normal neurologic status, normal ear-nose-throat inspection, and the stitched wound had a normal aspect. Laboratory investigations showed a slight leukocytosis (12.600 µl^−1^) with neutrophilia (85 %), but a normal CRP (6.6 mg l^−1^) and sedimentation rate (2 mm u^−1^). He was hospitalized for observation and neurologic monitoring, the latter remaining normal. Amoxicillin-clavulanic acid was continued.

**Fig. 1.**
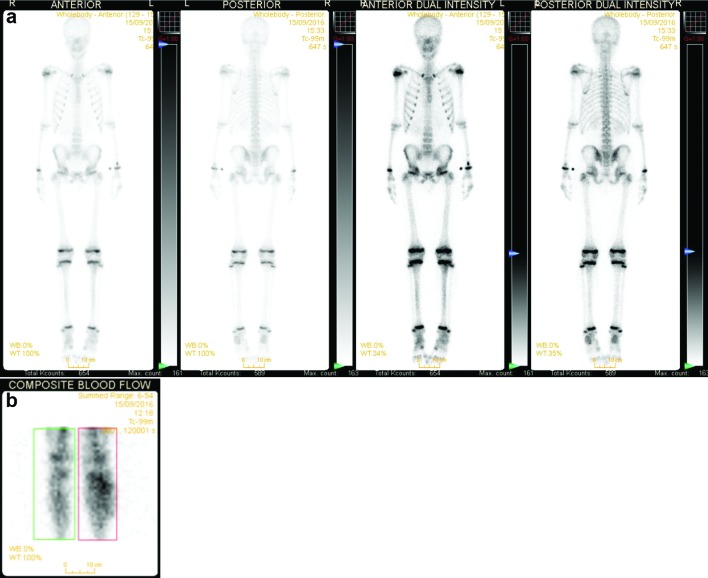
Results of bone scintigraphy, with (a) normal nucleotide signal distribution excluding osteomyelitis and septic arthritis, and (b) increased blood flow in the cranial part of the left lower leg with global swelling, indicating an important soft tissue inflammation.

The day after admission (day 2), he remained febrile (up to 38.9 °C). Examination now showed evacuation of some pus from his leg wound, with a slight swelling, redness and local pain. The pus was sent for culture on two separately collected cotton-tipped swabs (supplementary data S1). Ultrasound of the leg showed a small (11×3 mm) subcutaneous collection, but could not differentiate between residual blood and abscess. Bone scintigraphy showed no signs of osteomyelitis nor septic arthritis, but did show increased blood flow in the cranial part of the left lower leg with global swelling, indicating an important soft tissue inflammation (Fig. 1). Amoxicillin-clavulanic acid was increased to 100/10 mg kg^−1^d^−1^.

On day 3, cultures of the pus showed growth of *Aeromonas* species (on both samples), but susceptibility testing was not yet known. Clinically, there was some improvement with diminishing redness, but tenderness, swelling and pus evacuation continued. Antibiotic treatment was not altered.

On day 5, there were no additional pathogens found on culture and susceptibility testing of the *Aeromonas* species showed resistance to amoxicillin-clavulanic acid. The finding of *Aeromonas* species came unexpectedly. Only after he was actively asked, did he admit to having fallen into the ditchwater (stagnant river) after having injured his knee. Taking this into account, the *Aeromonas* species was considered to be the pathogen of his wound infection. Clinically, he had become afebrile since day 3 (thus 24 h after initiation of antibiotic therapy) and the redness had disappeared, but there was still some pus evacuation and a tender fluctuating swelling near the wound. Therefore, antibiotic treatment was switched to ciprofloxacin based on the antibiogram. Evolution of inflammatory parameters showed a swift normalization. On day 6, a control culture of the wound only showed growth of *Staphylococcus cohnii*, a contaminant. A blood culture taken on day 3 remained sterile.

On day 7, the cutaneous stitches were removed, because there was continuing evacuation of pus. Hereafter, the wound started to heal slowly. To improve wound healing, therapy with Bioptron lamp (polarized, polychromatic, non-coherent light) was started on day 8. He received two sessions of 9 min daily. For the rest of the time, the wound was covered with povidone-iodine 10 % compresses.

**Fig. 2.**
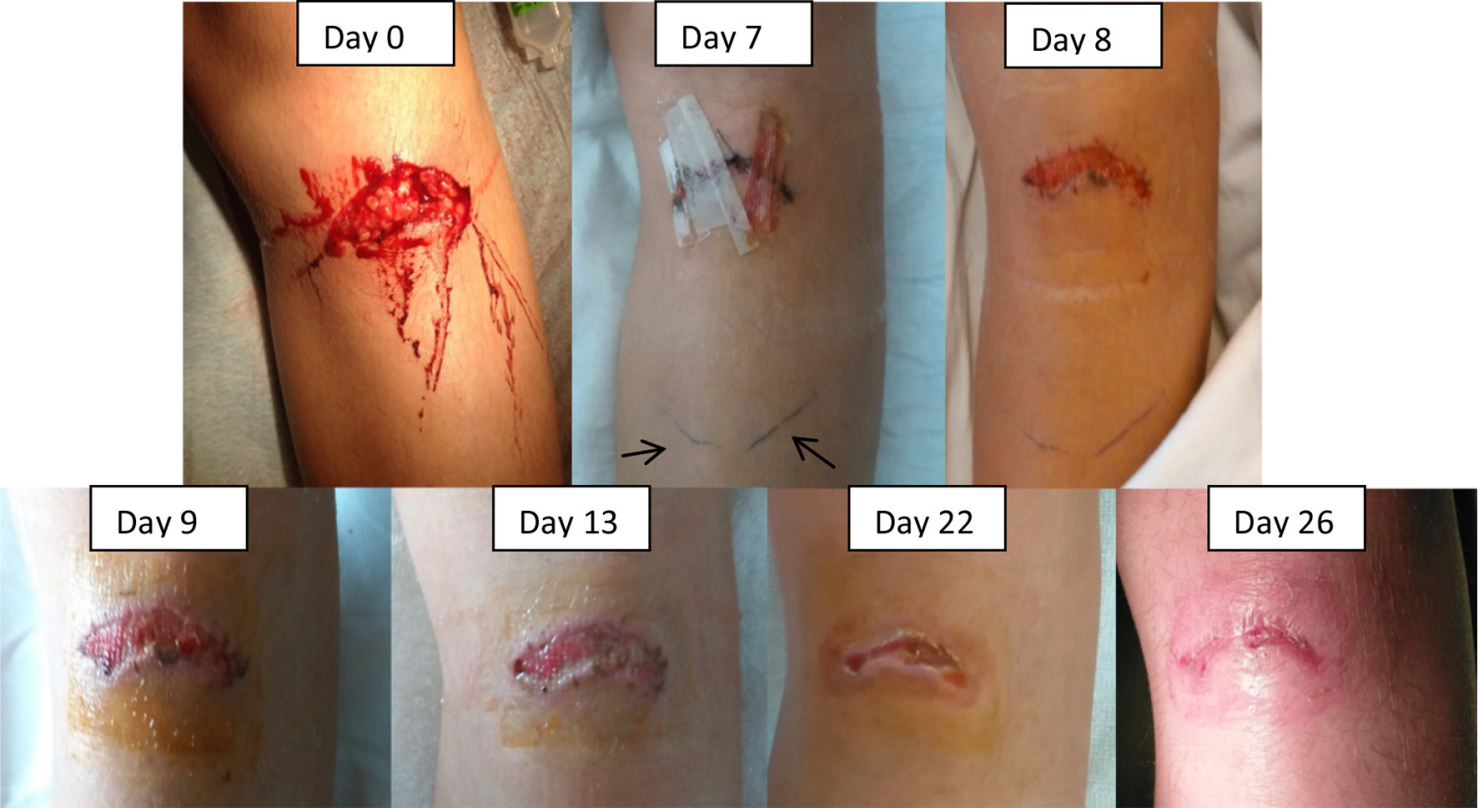
Pictures of the left lower leg, showing evolution over time. The pen marks on day 7 (indicated with arrows) show the line where redness had been at its maximum. On day 8, Bioptron lamp therapy was started, with afterwards a good granulation of the wound, starting at the edges. On day 26, a satisfying closure of the wound was achieved and Bioptron lamp treatment was stopped.

On day 13, after 7 days of intravenous treatment with ciprofloxacin, he was released home with continuation of ciprofloxacin orally for a total treatment duration of 14 days. Therapy with Bioptron lamp was continued in ambulatory setting (Two times 9 min with a 20 min interval, once daily), until satisfying wound healing on day 26 (Fig. 2).

## Discussion

We present a case of wound infection with *Aeromonas* in an otherwise healthy young boy. His history showed no chronic conditions, nor frequent infections. He had been infected with *Aeromonas* through contact with ditchwater in a rural area in Belgium.

Historically, *aeromonads* were initially recognized only as causing systemic illnesses in poikilothermic animals. Today, the genus *Aeromonas* is regarded not only as an important disease-causing pathogen of fish and other coldblooded species but also as the etiologic agent responsible for a variety of infectious complications in both immunocompetent and immunocompromised persons [1]. The infections are often severe in immunocompromised patients (cancer, cirrhosis, diabetes), leading to high rates of mortality [2–6]. *Aeromonas* infections in otherwise healthy persons are typically mild, although a severe infection with *Aeromonas* has been described in an otherwise healthy infant [7].

Gastroenteritis is the most common disease associated with *Aeromonas*. The second most common disease is wound infection. Wound infections with *Aeromonas* usually occur in previously healthy individuals following a penetrating or abrasion injury that occurs in an aquatic environment or soil where *Aeromonas* species are present in high numbers [1, 8]. The genus *Aeromonas* is considered to be almost synonymous with water and aquatic environments, being isolated from rivers, lakes, ponds, seawater (estuaries), drinking water, groundwater, wastewater, and sewage [1]. The more severe wound infections like necrotizing fasciitis or myonecrosis are most often seen in persons with immunodeficiency, liver disease or malignancy [1, 9, 10]. However, wound infections with *Aeromonas hydrophila* (*A. hydrophila*) can also be fatal in previously healthy individuals [8].

*Aeromonas sobria* (*A. sobria*) is the predominant human pathogen in the genus [11]. The species name *A. sobria* continues to be misused in publications. This species shares common phenotypes (aesculin, salicin, KCN, and l-arabinose negative) with the more common biovar of *Aeromonas veronii* (*A. veronii*), which is responsible for many human infections. What authors are incorrectly reporting as ‘*A. sobria*’ is in actuality *A. veronii* biovar sobria [1]. In our case, the laboratory of microbiology initially reported the strain as *A. sobria*. However, rechecking of the crude data shows in fact that the MALDI-TOF (Matrix assisted laser desorption/ionisation time-of-flight analyzer) results are consistent with an *Aeromonas* infection, but could not differentiate between *A. sobria* and *A. hydrophila*/*caviae*. In this case, the mentioned *A. sobria* does not concern the human pathogen *A. veronii* biovar sobria, but the true *A. sobria* which is not known to be pathogenic in humans. Therefore, it must be concluded that in our case the pathogen was most likely *A. hydrophila*. The antibiogram was not influenced by the indeterminate subtyping and is therefore reliable.

The first case of *Aeromonas* wound infection was in 1979, in a 19 year old man who had a minor leg injury while diving in river water [12]. Further reports have shown that trauma with water and/or dirt exposure are often associated with *Aeromonas* infections. They occur mostly in previously healthy individuals, and bacteremia or severe infections are rare in this group [13–15]. The chance of wound infections with *Aeromonas* species is higher during the warmer months of the year, since in those conditions *aeromonads* are found in increased concentrations in aquatic ecosystems [1]. The tsunami that struck Thailand in December 2004 resulted in many skin-and-soft-tissue infections. *Aeromonas* was the most common pathogen identified, accounting for 22.6 % of all isolates [16].

*Aeromonas* strains are almost universally susceptible to fluoroquinolones, and show high rates of resistance to other antibiotics [1, 2, 6, 13, 17]. Thus, *Aeromonas* infections should be best treated with fluoroquinolones [8]. In immunocompromised patients, fluoroquinolones should already be added to empiric treatment in case of a suspicion of necrotizing fasciitis [2]. Without fluoroquinolones, initial antibiotic treatment has proven to be inadequate in the majority of cases [14].

In our case, at first presentation no one spontaneously mentioned, nor was actively asked after trauma mechanism and circumstances. Therefore empiric treatment of his wound infection included only coverage for the usual skin colonizers. Only after the finding of the *Aeromonas* species, a repeated thorough history revealed that he had fallen into ditchwater. This emphasizes the importance of a thorough history taking at the emergency department.

Recommended intravenous therapy in classic skin and soft tissue infections is 3 days if there is clinical improvement [18]. We chose a longer course of intravenous treatment because of the unordinary pathogen and the absence of a treatment protocol for soft tissue infections with this pathogen.

Unfortunately, a first blood culture was only done 24 h after initiation of intravenous antibiotic treatment with amoxicillin-clavulanic acid. However, since pus cultures showed only *Aeromonas* species being resistant to amoxicillin-clavulanic acid and the patient was only taking amoxicillin-clavulanic acid, it is unlikely that blood cultures would have been positive if taken earlier. Moreover, there were never clinical nor biochemical signs of sepsis or bacteremia.

Under the use of polarized, polychromatic, non-coherent light therapy, there was swift and satisfying wound healing in this case. This therapy uses light with wavelengths from 480 to 3200 nm, with 95 % polarization using the principle of the Brewsters angle, and intensity of 2.4 J cm^-2^. It is known to have positive effects on healing of surgical wounds, burn wounds and ulcers, as well as ankle sprains and carpal tunnel syndrome [19–24]. It is believed to act by decreasing pro-inflammatory cytokines, increasing anti-inflammatory cytokines, increasing the T-helper lymphocyte population, increasing IgM and IgA, and decreasing immunocomplexes [25–27]. To our knowledge, this is the first reported case where polarized, polychromatic, non-coherent light therapy has been applied on an infected wound. Since there are no known adverse effects, only the size and amount of wounds may be a (practical) problem when considering this type of therapy (and of course availability). Adequate antibiotic therapy remains of course the mainstay of treating infectious wounds, but light therapy may be an additional therapy. Further research must be performed to prove its superiority over spontaneous healing.

### Conclusion

This case illustrates the importance of taking a thorough history when faced with a patient with wounds and wound infections. It is essential to ask for the circumstances and mechanism of the injury, since they are indicative of the pathogens that should be taken into consideration when selecting empiric antibiotic treatment. In case of penetrating injuries with water and/or dirt exposure, addition of a quinolone is necessary to cover *Aeromonas* species.

In our case, there was satisfying wound healing after initiation of polarized, polychromatic, non-coherent light therapy. This therapy may be an additional option for improved healing of infectious wounds, but more research is needed.
